# Carbon-Induced Structural Evolution and Synergistic Enhancement of Wear and Corrosion Resistance in (AlFeCoNi)C High-Entropy Alloy Carbide Films

**DOI:** 10.3390/ma18184411

**Published:** 2025-09-22

**Authors:** Duoli Chen, Yefeng Zhou, Xianting Yang, Mengyuan Guo, Jun Liang, Deming Huang, Yu Ni, Yurong Zhou, Yantao Li, Xin Jiang

**Affiliations:** 1 School of Intelligent Manufacturing, Mianyang Teachers’ College, Mianyang 621000, China; 18780000611@163.com (D.C.); 18181696545@163.com (Y.Z.); yxt20240223cj@163.com (X.Y.); 18208225926@163.com (M.G.); liangjuntomato@163.com (J.L.); deming_huang03@163.com (D.H.); niyuheigeer@163.com (Y.N.); zhouyurongpzh@163.com (Y.Z.); 2 Key Laboratory of Advanced Technologies of Materials, Ministry of Education, School of Materials Science and Engineering, Southwest Jiaotong University, Chengdu 610031, China

**Keywords:** high-entropy alloy films, carbon doping, structural transition, tribological properties, corrosion resistance

## Abstract

The (AlFeCoNi)C high-entropy alloy carbide films (HECFs) with tunable carbon contents were fabricated by magnetron sputtering to investigate the carbon-driven structural evolution and its coupling effects on mechanical and chemical properties. With increasing carbon incorporation (0–47.6 at.%), the HECFs formed a composite structure of amorphous phase and BCC nanocrystalline phase, as evidenced by XRD and TEM. Atom probe tomography (APT) reveals Al segregation in the film. Remarkably, the wear rate decreases exponentially from 4.8 × 10^−5^ to 6.7 × 10^−6^ mm^3^/N·m, attributed to the amorphous carbon phase acting as solid lubricant. Simultaneously, the corrosion current density reduces by two orders of magnitude (7.2 × 10^−8^ A/cm^2^ in 3.5% NaCl), benefiting from the amorphous network inhibiting ion diffusion pathways. This work establishes a carbon-content–property correlation paradigm for designing multifunctional HEA films in extreme environments.

## 1. Introduction

High-entropy alloy films (HEAFs) have emerged as promising candidates [1,2,3] for extreme-environment applications, such as aerospace components, marine engineering systems, and nuclear reactors, where materials are subjected to simultaneous mechanical wear and corrosive degradation. While conventional HEAFAs exhibit exceptional strength and thermal stability, their performance in tribological and chemically aggressive environments remains constrained by intrinsic limitations: single-phase structures often lack adaptive lubrication mechanisms, while grain boundaries and defects in crystalline phases provide pathways for corrosive ion penetration. Addressing this dual challenge requires innovative strategies to decouple structural design from property trade-offs.

AlFeCoNi-based high-entropy thin films [4,5] exhibit outstanding comprehensive mechanical properties, combining high hardness with high toughness. However, they still face significant challenges in corrosion resistance and wear resistance. Currently, researchers primarily enhance their performance through alloying. Goodelman et al. [6] investigated the influence of Ti and Al atomic content on the properties of thin films in the AlCoCrFeNiTi system. They found that Al and Ti, as “large atomic radius elements” (>140 pm), competitively drive phase formation. Ti content exceeding 7 at.% induces amorphization, while below this threshold, Al promotes an ordered BCC structure (with high Al content leading to enhanced crystallinity). Wu et al. [7] studied FeCoNiAlCu_x_ high-entropy alloy coatings, and found that the introduction of Cu atoms enhances both the wear resistance and corrosion resistance of the coatings. Due to the friction-reducing effect of copper-rich oxides, the lowest friction coefficient of the coating was 0.381, and the smallest wear volume reached 0.095 mm^3^. Owing to the formation of a copper-rich passive film, the lowest corrosion current density was 1.08 × 10^−4^ A/cm^2^. Although the introduction of alloying elements such as copper (Cu) and titanium (Ti) can improve the mechanical properties and corrosion resistance of the coating to a certain extent, it still falls short of meeting the requirements for extreme working conditions.

Numerous literature studies [8,9,10,11] demonstrate that doping with non-metallic atoms can also effectively enhance the mechanical properties of the films while simultaneously improving their corrosion resistance. Li et al. [10] introduced C atoms into the VNbMoTaW high-entropy system via reactive magnetron sputtering, and found that carbon doping promoted the formation of an amorphous carbon phase, significantly enhancing the corrosion resistance of the films, which is superior to that of 316L stainless steel. Fritze et al. [11] introduced carbon atoms into CrNbTaTiW films via reactive magnetron sputtering and found that C doping induced grain refinement and formed a supersaturated solid solution, which significantly enhanced the hardness and crack resistance of the films.

Herein, we propose a carbon-driven structural engineering approach to tailor the multifunctionality of (AlFeCoNi)C_x_ High-entropy carbide films (HECFs). The (AlFeCoNi)C_x_ HECFs are targeted for applications in extreme environments such as aerospace, marine engineering, and nuclear systems, where materials are subjected to simultaneous mechanical wear and corrosion. These applications require a combination of excellent wear resistance and superior corrosion resistance, which are often mutually exclusive in conventional materials. High-power pulsed magnetron sputtering (HPPMS) is selected for depositing the (AlFeCoNi)C_x_ films due to its unique advantages: (1) Precise control over composition and microstructure through C_2_H_2_ flow regulation. (2) High ionization efficiency, leading to dense and well-adhered films with reduced defects. (3) Ability to deposit at room temperature, avoiding substrate thermal degradation. (AlFeCoNi)C_x_ HECFs are prepared via HPPMS using the AlFeCoNi spliced metal target at various C_2_H_2_ flow rates (F_c_). The effects of carbon atom incorporation on the phase structure, mechanical properties, and corrosion resistance of (AlFeCoNi)C_x_ HECFs are systematically investigated. The objective of this study is to offer insights into the structure, mechanical properties, and corrosion resistance of HECFs.

## 2. Materials and Methods

**Figure 1:**
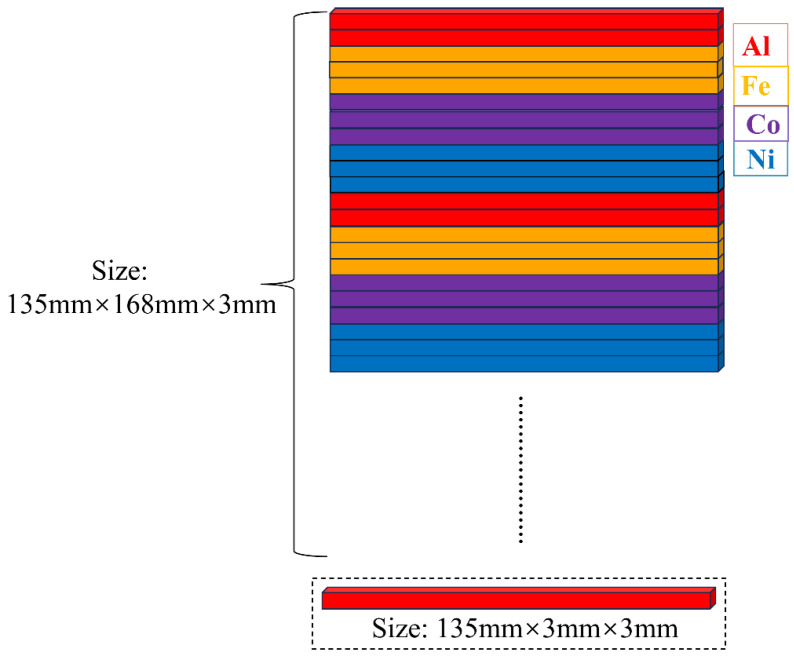
The schematic diagram of metal splicing target.

(AlFeCoNi)C_x_ HECFs were deposited on single-crystal Si (100) and 304 stainless steel substrates (304 SS) via high-power pulsed magnetron sputtering (HPPMS) using a four-cathode unbalanced magnetron system [12]. As shown in Figure 1, the sputtering target consisted of high-purity (≥99.9%) Al, Fe, Co and Ni rods spliced into a 168 mm × 135 mm × 3 mm composite assembly. The 304 SS were mechanically polished using SiC abrasive paper up to 1200 grit, followed by diamond paste polishing to a mirror finish. Then, substrates were cleaned ultrasonically for 20 min in acetone and ethanol, followed by mounting in the chamber. After achieving a base pressure of 1 × 10^−3^ Pa, 40 sccm Ar was introduced into the chamber, and the target and substrate pre-cleaning was performed by plasma sputtering for 10 min and 20 min, respectively. During film growth, Ar flow was maintained at 40 sccm while C_2_H_2_ flow rates were varied at 0, 3 and 5 sccm. The target was powered by an HPPMS supply (HPP12S1, Chengdu Pulse Tech, Chengdu, China) operating at 800 V, 150 μs pulse width, and 200 Hz frequency, with a concurrent −50 V substrate bias. The detailed deposition parameters are listed in Table 1.

The composition and morphologies of the (AlFeCoNi)C_x_ HECFs were obtained by a field emission scanning electron microscope (FESEM, Sigma 360, Carl Zeiss AG, Oberkochen, Germany) equipped with Energy dispersive spectroscopy (EDS, Oxford Xplore 30, Oxford Instruments, Abingdon, UK). Crystalline structure was analyzed by X-ray diffraction (XRD, Ultima IV, Rigaku, Tokyo, Japan) and transmission electron microscopy (TEM, FEI Talos F200S, Thermo Fisher Scientific, Branford, CT, USA). The structural information of carbon in the film was characterized using a Raman spectrometer (Raman, LabRAMHR Evolution, HORIBA Scientific, Paris, France) with an excitation wavelength of 532 nm. Near-atomic-scale elemental distribution was investigated via atom probe tomography (APT, LEAP^™^ 5000XR, CAMECA, Paris, France). The tribological properties of the HECFs were tested by a tribometer (CSEM, Neuchatel, Switzerland) with Al_2_O_3_ balls of diameter 6 mm as friction pairs at a load of 2 N in air. The wear is reciprocating, with a stroke of 6 mm and a set wear cycle of 1000 r (wear distance = 1000 × 2 × 6 mm). The morphologies of the wear tracks were observed by a field emission scanning electron microscope (FE-SEM, Sigma 360, Carl Zeiss AG, Oberkochen, Germany). The corrosion behavior of the HECFs was evaluated in 3.5 wt.% NaCl solution through potentiodynamic polarization and electrochemical impedance spectroscopy (EIS, CorrTest CS350H workstation, Wuhan Corrtest Instrument Co., Ltd., Wuhan, China) using a three-electrode cell: saturated calomel reference electrode (SCE), platinum counter electrode, and specimen working electrode. Potentiodynamic polarization scans employed a ±0.5 V potential window relative to open-circuit potential at 1 mV/s. Electrochemical impedance spectroscopy (EIS) measurements were performed over a frequency range of 100 kHz to 10 mHz with an AC amplitude of 10 mV. The electrolyte was maintained at 25 ± 1 °C and deaerated by purging with high-purity argon (100 cm^3^/min) for 30 min under mechanical stirring (500 rpm). Experiments commenced once the dissolved oxygen concentration dropped below 0.1 ppm. All samples were polished to a mirror finish. The acquired EIS data were subsequently analyzed by the equivalent circuit modeling software Zview (version 2.70, Scribner Associates Inc., Southern Pines, NC, USA) to extract quantitative electrochemical parameters. Both wear tests and electrochemical tests were conducted three times, and representative data were selected for plotting.

## 3. Results

### 3.1. Composition of the (AlFeCoNi)C_x_ HECFs

**Figure 2:**
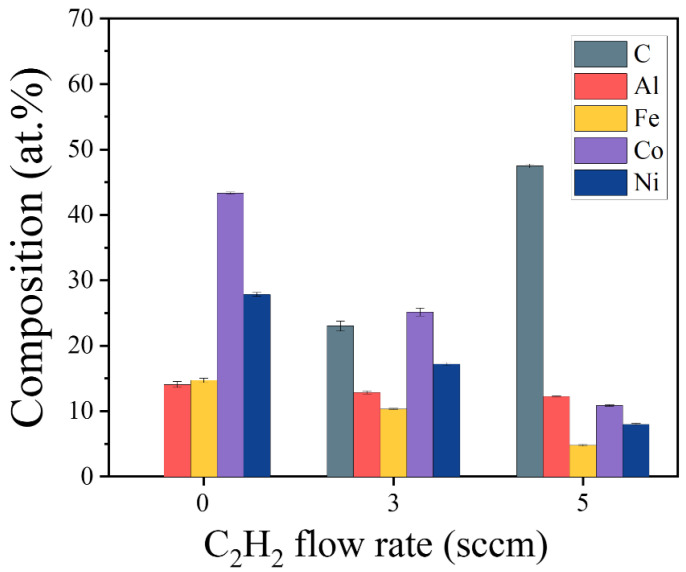
The composition of the (AlFeCoNi)C_x_ HECFs deposited at different F_c_.

Figure 2 depicts the composition of (AlFeCoNi)C_x_ HECFs synthesized at varying F_c_. As the F_c_ increases from 0 to 5 sccm, the carbon content rises progressively from 0 at.% to 23.0 at.% and 47.6 at.%, respectively, indicating successful incorporation of graded carbon concentrations. Concurrently, corresponding reductions occur in metallic constituents: Al decreases from 14.1 at.% to 12.1 at.%, Fe from 14.7 at.% to 4.7 at.%, Co from 43.6 at.% to 10.8 at.%, and Ni from 27.9 at.% to 8.1 at.%. These trends collectively demonstrate precise carbon stoichiometry control in (AlFeCoNi)C_x_ HECFs through C_2_H_2_ flow regulation, establishing a foundation for probing carbon-dependent microstructure–property relationships.

### 3.2. Microstructure of the (AlFeCoNi)C_x_ HECFs

**Figure 3:**
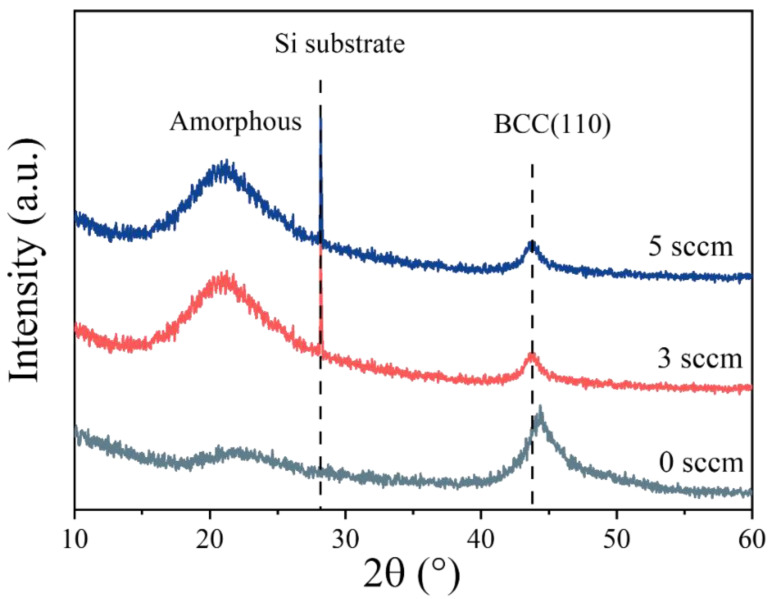
XRD spectra of the (AlFeCoNi)C_x_ HECFs deposited at different F_c_.

Figure 3 presents the XRD patterns of the (AlFeCoNi)C_x_ HECFs. All samples exhibit characteristic peaks corresponding to the amorphous phase (approximately 21.7°) and the body-centered cubic (BCC) phase (approximately 44.3°). The curves for 3 sccm and 5 sccm show an additional sharp diffraction peak around 28°, which corresponds to the silicon (Si) substrate. As the F_c_ increases, the intensity of the amorphous peak at 21.7° gradually enhances, while the peak at 44.3° (the (110) plane of the BCC phase) gradually weakens. This indicates that carbon incorporation induces a structural evolution in the film, leading to an increase in the content of the amorphous phase and a decrease in the content of the BCC phase. The above results demonstrate that variations in carbon content significantly affect the phase structure of the (AlFeCoNi)C_x_ HECFs. Similar results have also been found in the (CrHfMoTaW)C system [13]. These structural differences, arising from different levels of carbon incorporation, are clearly observable through XRD analysis.

**Figure 4:**
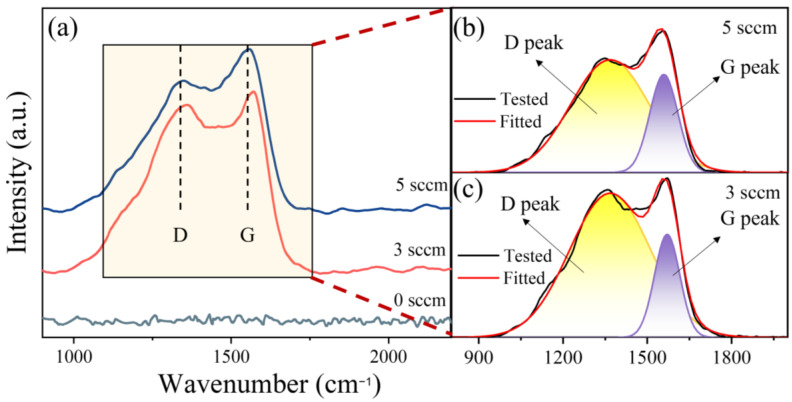
Raman spectra of the (AlFeCoNi)C_x_ HECFs with different F_c_. (**a**) Raman spectra of the tested HECFs; (**b**) Fitted spectra of HECFs deposited at F_c_ = 5 sccm; (**c**) Fitted spectra of HECFs deposited at F_c_ = 3 sccm.

Figure 4a displays the Raman spectra of (AlFeCoNi)C HECFs synthesized with varying F_c_. The spectrum of the film deposited at 0 sccm exhibits no discernible D or G peaks, indicating the absence of an amorphous carbon (a-C) phase. With the introduction of carbon, characteristic vibrational modes emerge at approximately 1370 cm^−1^ (D-peak) and 1560 cm^−1^ (G-peak), unambiguously confirming a-C phase formation within the HECFs. Detailed peak fitting analysis (Figure 4b,c) reveals the following specific peak positions: D-peaks at 1367 cm^−1^ (3 sccm) and 1366 cm^−1^ (5 sccm), paired with G-peaks at 1570 cm^−1^ (3 sccm) and 1558 cm^−1^ (5 sccm), respectively. The full width at half maximum (FWHM) values for D-peaks measure 339 cm^−1^ (3 sccm) and 328 cm^−1^ (5 sccm), while G-peaks exhibit narrower FWHM of 106 cm^−1^ (3 sccm) and 117 cm^−1^ (5 sccm) under corresponding conditions. Notably, the spectral features remain stable across different F_c_, with minimal peak shifting. The I_D_/I_G_ ratio, a well-established indicator of sp^2^-carbon clustering [14,15], systematically decreases from 4.49 to 3.21 with increasing F_c_, demonstrating a reduction in sp^2^ bonding and an increase in sp^3^ bonding at higher carbon concentrations.

**Figure 5:**
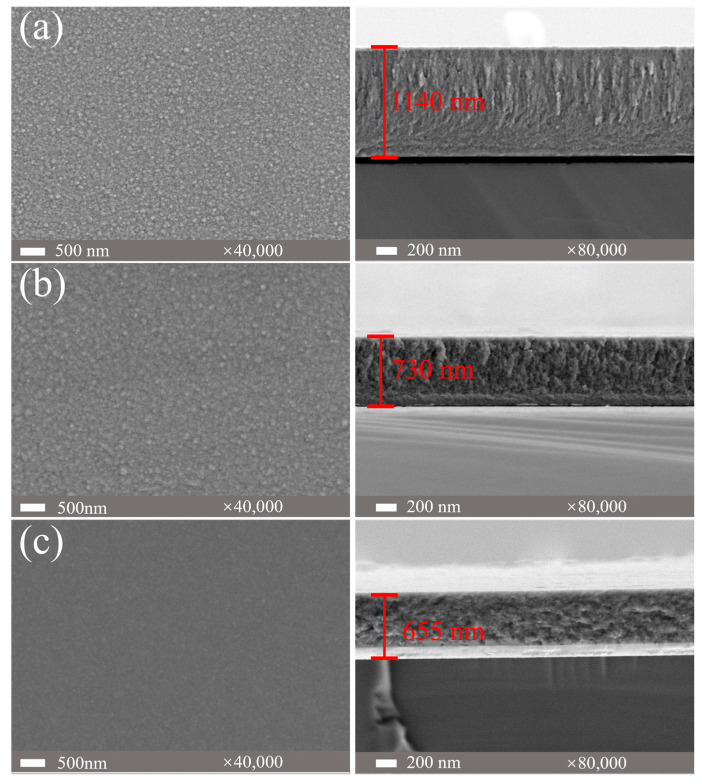
Surface and cross-sectional morphologies of (AlFeCoNi)C_x_ HECFs with different F_c_: (**a**) 0 sccm, (**b**) 3 sccm, (**c**) 5 sccm.

Figure 5 presents the surface and cross-sectional morphologies of (AlFeCoNi)C_x_ HECFs with varying F_c_. The surface morphology exhibits a distinct smoothing effect with increasing carbon content. The film deposited at F_c_ = 0 sccm (Figure 5a) displays numerous granular bulges and a coarse topography. With F_c_ increases to 3 sccm (Figure 5b), the surface roughness is notably reduced, as evidenced by the decreased density and size of granular features. At the F_c_ = 5 sccm (Figure 5c), the film exhibits a smooth and nearly featureless surface, indicating a transition to a more homogeneous microstructure. More significantly, carbon incorporation induces substantial alterations in the cross-sectional microstructure. The film deposited at F_c_ = 0 sccm exhibits a well-defined columnar structure. This columnar growth mode becomes increasingly suppressed at F_c_ = 3 sccm. At F_c_ = 5 sccm, the cross-section of the film develops a markedly roughened morphology. Simultaneously, film thickness was observed to decrease gradually with increasing carbon content. This phenomenon is primarily attributed to the “target poisoning” effect [16,17], where adsorption of acetylene gas on the target surface hinders atomic sputtering [18], ultimately reducing the sputtering yield.

**Figure 6:**
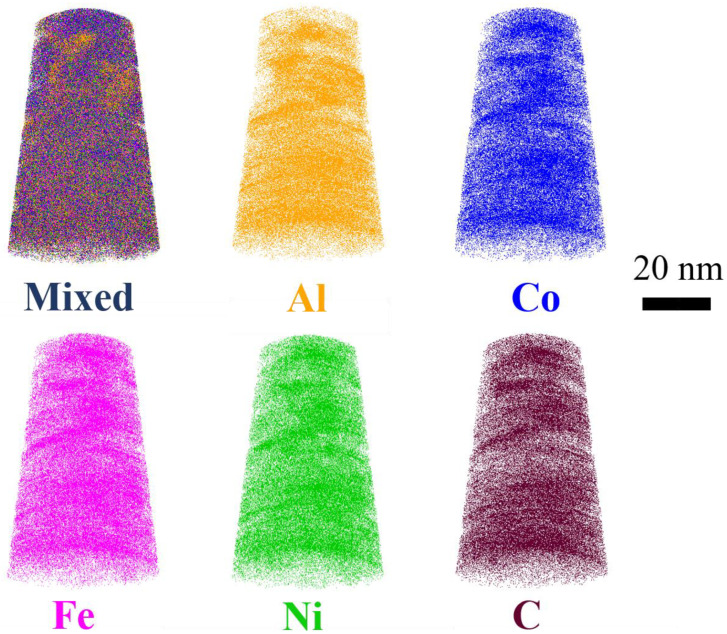
Elemental distribution of (AlFeCoNi)C_x_ HECFs deposited at F_c_ = 3 sccm. Three-dimensional reconstruction of an APT dataset, and atom maps of mixed, Al, Fe, Co, Ni and C.

Figure 6 presents the individual and mixed atom maps of Al, Fe, Co, Ni, and C. While Fe, Co, Ni and C exhibit homogeneous distributions without detectable segregation, Al displays pronounced segregation behavior. This phenomenon can be attributed to the strong aggregation tendency of aluminum atoms, which is commonly observed in multicomponent systems [19,20,21] due to its relatively low mixing enthalpy with transition metal elements.

**Figure 7:**
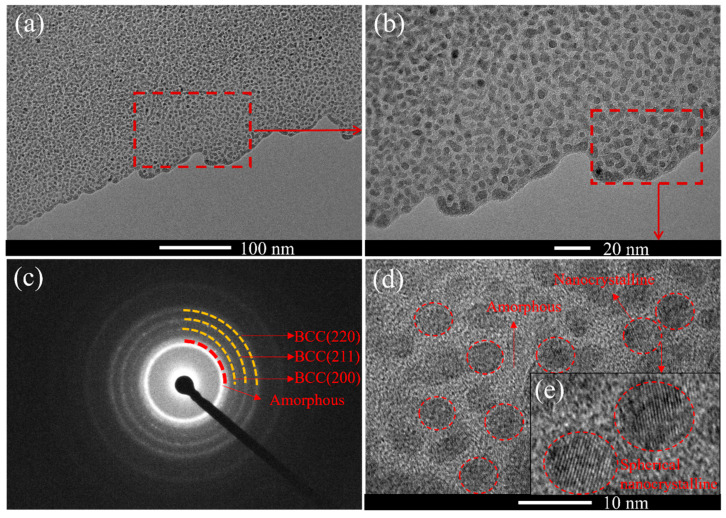
TEM images of (AlFeCoNi)C_x_ HECFs deposited at F_c_ = 3 sccm, (**a**) low magnified images, (**b**,**d**,**e**) are high resolution images, (**c**) selected area electron diffraction pattern.

Figure 7 presents the transmission electron microscopy (TEM) analysis results of the (AlFeCoNi)C_x_ HECFs prepared with F_c_ = 3 sccm. Figure 7a is the macrostructure of the HECFs, showing obvious light and dark contrast. Figure 7b is a magnified view of the red-dashed-box region in Figure 7a, revealing a large number of black spherical areas surrounded by white areas. Figure 7c shows the selected area electron diffraction (SAED) pattern of the HECFs. Distinct diffraction rings are visible. The inner diffuse halo region corresponds to an amorphous phase, while the outer diffraction rings correspond to crystal planes of a BCC structure, specifically BCC (200), BCC (211), and BCC (220), which is consistent with the XRD results. This indicates the coexistence of both amorphous and nanocrystalline phases within the HECFs. Figure 7d,e provide a higher magnification view of the microstructure. White regions correspond to the amorphous structure, while black regions represent the nanocrystalline phase. This result is similar to the microstructure of (FeCoCrNi)NC_x_ films [22]. These results demonstrate that the (AlFeCoNi)C_x_ HECFs possess a composite structure consisting of an amorphous matrix and spherical BCC nanocrystals approximately 10 nm in diameter.

The formation of this amorphous + nanocrystalline composite structure is closely related to the introduction of C atoms. According to reference [23], the mixing enthalpies of C with Al, Fe, Co, and Ni are −36, −50, −42, and −39 kJ/mol, respectively, which are significantly higher than the mixing enthalpies of refractory metal elements with C (such as Ti-C: −109 kJ/mol, Nb-C:−102 kJ/mol). Generally, the more negative the mixing enthalpy, the higher the atomic bonding energy, and conversely, the weaker the bonding [24,25]. Therefore, the binding energy between C and Al, Fe, Co, Ni is weak and cannot form covalent bonds. Therefore, it can be inferred that the introduced C atoms initially dissolve in the gaps of the BCC structure, and after the gaps become saturated with solid solution, form an amorphous carbon phase on the outside, ultimately forming a composite structure of amorphous (including amorphous carbon phase and amorphous metal phase) + BCC nanocrystals.

### 3.3. Tribological Properties of the (AlFeCoNi)C_x_ HECFs

**Figure 8:**
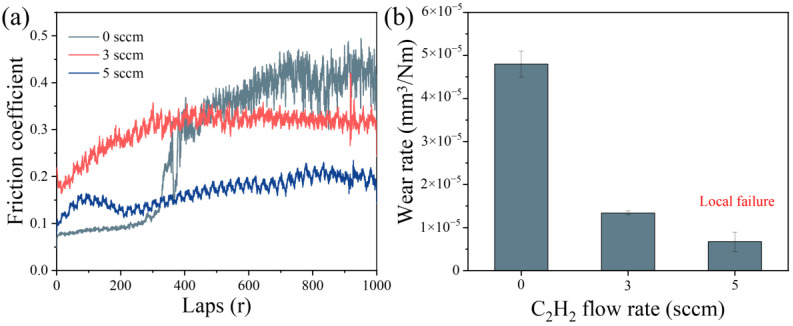
(**a**) Friction coefficient curves, (**b**) wear rates of (AlFeCoNi)C_x_ HECFs deposited at different carbon flow rates.

Figure 8a presents the friction coefficient curves of (AlFeCoNi)C_x_ HECFs deposited at different F_c_. The HECFs-0 sccm sample exhibits significant fluctuations in friction coefficient, indicating film failure during sliding. With increasing carbon content, the friction coefficients stabilize at lower values of ~0.3 (HECFs-3 sccm) and ~0.2 (HECFs-5 sccm), demonstrating improved tribological performance. This reduction is primarily attributed to the formation of lubricious amorphous carbon phases within the films. As shown in Figure 8b, the wear resistance of (AlFeCoNi)C_x_ films shows a strong dependence on carbon content. The wear rate decreases remarkably from 4.8 × 10^−5^ mm^3^/N·m (HECFs-0 sccm) to 1.3 × 10^−5^ mm^3^/N·m (HECFs-3 sccm) and then to 6.7 × 10^−6^ mm^3^/N·m (HECFs-5 sccm). This enhancement in friction stems from two synergistic effects: (1) the lubricating properties of amorphous carbon phases and (2) the strengthening effect of the nanocomposite structure. These results demonstrate that carbon incorporation effectively transforms the AlFeCoNi system into a high-performance tribological film.

**Figure 9:**
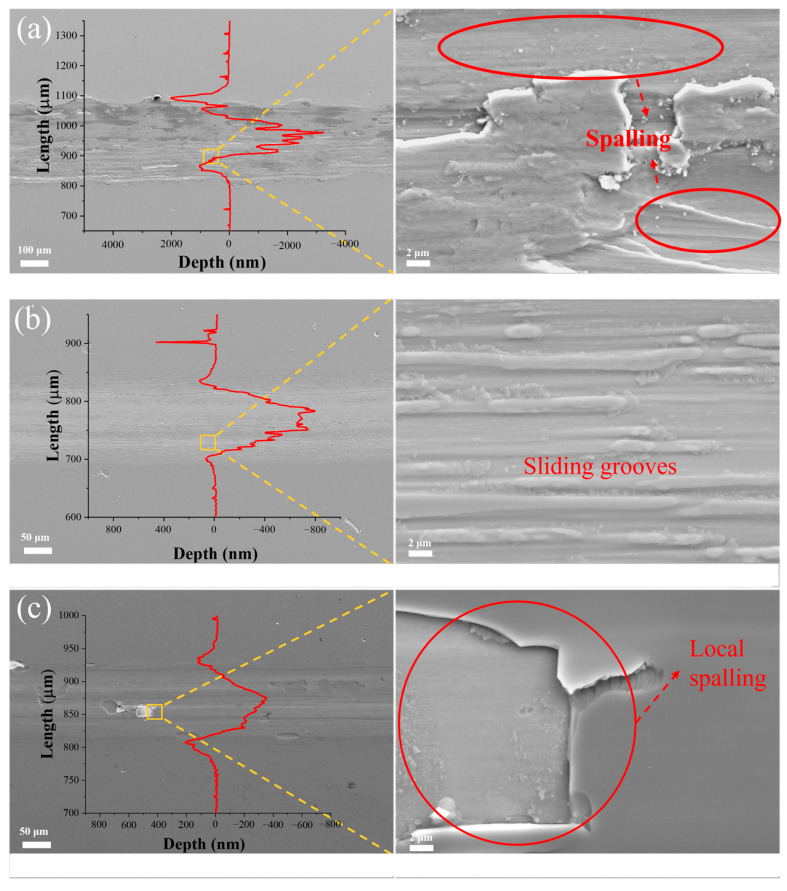
Wear track profiles and morphologies of (AlFeCoNi)C_x_ HECFs deposited at F_c_ = (**a**) 0 sccm, (**b**) 3 sccm, and (**c**) 5 sccm.

Figure 9 presents the wear track profiles and morphologies of (AlFeCoNi)C_x_ HECFs fabricated under varying F_c_. As illustrated in Figure 9a, the (AlFeCoNi)C_x_ HECFs without carbon incorporation exhibits show a very rough morphology and extensive spalling. Moreover, the profile of the abrasion shows that the deepest point is close to 3 μm, exceeding the thickness of the film (1.1 μm), indicating that the film has failed. With the F_c_ increase to 3 sccm, the wear tracks demonstrate a characteristic sliding grooves, suggesting that the wear mechanism is abrasive wear. the (AlFeCoNi)C_x_ HECFs deposited at F_c_ = 5 sccm show the shallowest wear profile, but there is obvious spalling in some areas, indicating that the brittleness of the film increases with further increase in carbon content. The spalling and delamination observed for the HECFs-5 sccm film (Figure 9c) are indicative of adhesion failure, likely driven by a combination of high intrinsic stress and interface weakening [22]. This phenomenon indicates a carbon-dependent embrittlement mechanism, where progressive carbon enrichment significantly increases brittleness, thereby drastically amplifying the risk of film structural failure.

### 3.4. Corrosion Resistance of the (AlFeCoNi)C_x_ HECFs

**Figure 10:**
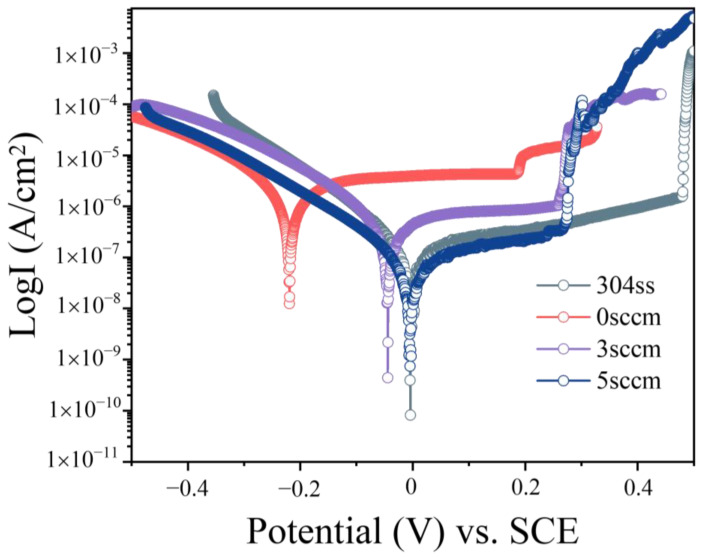
Potentiodynamic polarization curves of bare 304 SS and the (AlFeCoNi)C_x_ HECFs in 3.5 wt.% NaCl.

Figure 10 presents the potentiodynamic polarization curves of the deposited (AlFeCoNi)C_x_ HECFs, along with the bare substrate as a reference. The corrosion potential (E_corr_) and corrosion current density (I_corr_) were derived from Tafel extrapolation, with the results summarized in Table 2. Since I_corr_ is directly proportional to the corrosion rate [26], a lower value signifies enhanced corrosion resistance. A clear trend is observed where the corrosion resistance of the HECFs improves progressively with increasing F_c_. Remarkably, the HECFs deposited at 5 sccm exhibits the lowest anodic current density (7.16 × 10^−8^ A/cm^2^), indicating superior corrosion resistance compared to other samples. This enhancement is primarily attributed to the formation of an amorphous-dominated structure at higher carbon content, as amorphous materials inherently possess fewer defect sites [27] (e.g., grain boundaries and dislocations) that typically act as initiation points for corrosion [28].

**Figure 11:**
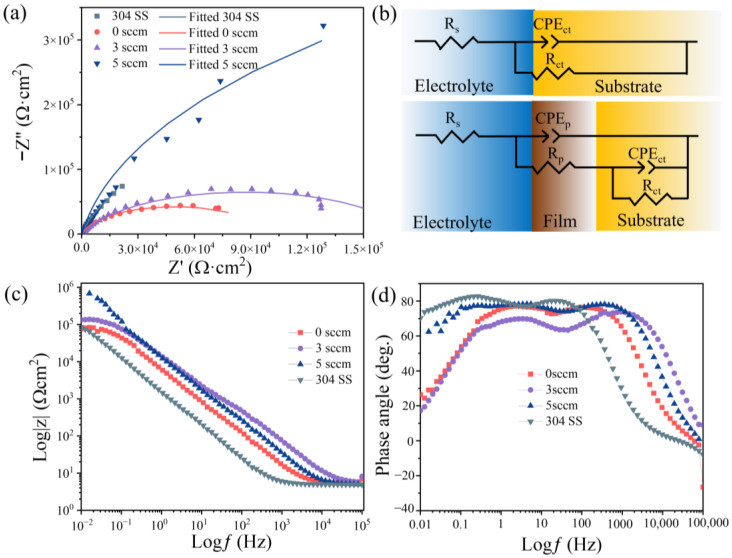
(**a**) Nyquist plots from EIS data of 304 SS and the (AlFeCoNi)C_x_ HECFs, (**b**) equivalent circuit model of the substrate and HECFs coated substrate, (**c**,**d**) Bode plots.

Figure 11a presents the Nyquist plots of the deposited films along with 304 stainless steel (304 SS) for comparison. All samples exhibit semicircular arcs. As the carbon flow rate increases, the semicircle diameters systematically increase, reaching its maximum at 5 sccm. The fitted parameters, including the goodness of fit (χ^2^, where lower values indicate better agreement), are summarized in Table 3. The equivalent circuit model shown in Figure 11b consists of R_s_ (solution resistance), R_p_ (film resistance), and R_ct_ (charge transfer resistance). Due to surface inhomogeneity, constant phase elements (CPEs) were employed instead of ideal capacitors: CPE_p_ represents the film response, while CPE_ct_ corresponds to the substrate interface. The semicircle diameters in the Nyquist plots quantitatively represent R_ct_, with larger diameters indicating higher charge transfer resistance and thus improved corrosion resistance. Notably, the film deposited at F_c_ = 5 sccm flow rate exhibits the highest R_ct_ value, slightly exceeding that of the 304 SS. The corrosion resistance was evaluated by extracting key parameters from the low-frequency (0.01 Hz) region, specifically the impedance modulus |Z|. Figure 11c reveals that the F_c_ = 5 sccm sample achieved the maximum |Z| value, confirming its superior performance compared to other samples. The above results confirm its superior corrosion resistance, consistent with the microstructural evolution observed in Figure 4. The 5 sccm condition produces a more homogeneous, predominantly amorphous phase with fewer defects. The trend in corrosion resistance follows the order: 5 sccm ≥ 304 SS > 3 sccm > 0 sccm, directly linking electrochemical performance to deposition parameters. The slightly higher chi-squared (χ^2^) value for the 5 sccm sample (0.0203) in the EIS fitting, compared to other samples, is noted. This is attributed to the exceptionally large and stable impedance response of this highly corrosion-resistant film, where even minimal instrumental noise becomes proportionally more significant in the goodness-of-fit calculation. Importantly, the key parameter extracted from the fit, the charge transfer resistance (R_ct_), which is orders of magnitude larger for this sample, is robust and is consistently supported by the potentiodynamic polarization results (Table 2).

## 4. Discussion

The incorporation of carbon fundamentally reconstructs the microstructure of AlFeCoNi-based films. At F_c_ = 0, the film exhibits a coarse columnar BCC structure (Figure 5a), consistent with conventional high-entropy alloy films [29,30]. With increasing carbon flux (up to 47.6 at.%), XRD (Figure 3) and TEM (Figure 7) conclusively demonstrate a transition to an amorphous matrix embedded with BCC nanocrystals (~10 nm). This dual-phase evolution arises from two synergistic effects: (1) Solid Solution Saturation: Weak mixing enthalpies between C and metallic elements (Al: −36 kJ/mol; Fe: −50 kJ/mol; Co: −42 kJ/mol; Ni: −39 kJ/mol) initially permit carbon dissolution into BCC interstices. (2) Amorphous Phase Separation: Upon exceeding solubility limits, excess carbon segregates to form an amorphous carbon (a-C) phase (Raman-confirmed D/G peaks in Figure 4), simultaneously disrupting long-range metallic ordering and promoting amorphous metal phase formation.

APT analysis (Figure 6) further reveals Al segregation, attributed to its low mixing enthalpy with transition metals. This nanoscale heterogeneity likely enhances interfacial strengthening but may locally compromise corrosion resistance—a trade-off requiring future optimization.

The exponential reduction in wear rate (from 4.8 × 10^−5^ to 3.9 × 10^−6^ mm^3^/N·m, Figure 8b) stems from three carbon-activated mechanisms: (1) Solid Lubrication: a-C phases (sp^2^/sp^3^ ratio decreasing with F_c_, Figure 4b,c) form shear-adaptive transfer films at sliding interfaces, reducing friction coefficients from 0.5 to 0.2 (Figure 8a). (2) Nanocomposite Strengthening: BCC nanocrystals (Figure 7d,e) impede dislocation motion and crack propagation, enhancing load-bearing capacity. Surface Smoothing: Carbon suppresses columnar growth (Figure 5), reducing asperity contact and abrasive grooving (Figure 9c vs. Figure 9a). Notably, at F_c_ = 5 sccm (47.6 at.% C), brittle spalling emerges (Figure 9c), indicating a critical carbon threshold beyond which embrittlement outweighs lubrication benefits. This suggests an optimal carbon window (e.g., ~23–40 at.%) for balanced toughness and wear resistance.

The corrosion current density (I_corr_) plunges by two orders of magnitude (to 7.16 × 10^−8^ A/cm^2^, Table 2) at high carbon content, outperforming 304 SS. EIS analysis (Figure 11, Table 3) confirms this via charge transfer resistance (R_ct_) maximization (1,062,900 Ω·cm^2^ at F_c_ = 5 sccm). This result is mainly attributed to the defect elimination and passive film stability. Amorphous networks eliminate grain boundaries and dislocation pathways (Figure 5c cross-section), blocking Cl^−^ ion diffusion [31,32,33,34]. The dense, carbon-rich surface (Figure 5c) impedes anodic dissolution, shifting E_corr_ nobly (−0.004 V_SCE_ at F_c_ = 5 sccm vs. −0.222 V_SCE_ at F_c_ = 0 sccm). Consequently, an optimal carbon content window is identified around approximately 23 at.% (F_c_ = 3 sccm). This composition achieves a synergistic enhancement in wear and corrosion resistance—reducing the wear rate by an order of magnitude and the corrosion current density by a factor of four compared to the undoped film—while effectively avoiding the brittle spalling behavior that compromises the performance of the film with 47.6 at.% (F_c_ = 5 sccm) carbon. The synergistic enhancement of wear and corrosion resistance achieved in the (AlFeCoNi)C_x_ HECFs, particularly at the optimal carbon content of ~23 at.%, suggests their high potential for application in extreme service environments. Specifically, these films are promising candidates for protecting critical components in marine engineering (e.g., bearings and seals in turbines and pumps), where materials are simultaneously subjected to mechanical degradation and corrosive attacks.

## 5. Conclusions

This study successfully fabricated (AlFeCoNi)C_x_ high-entropy alloy carbide films with carbon contents ranging from 0 to 47.6 at.% via HPPMS. The key experimental results are as follows:

1. XRD and TEM analyses confirmed a carbon-induced microstructural transition from a columnar BCC structure to a nanocomposite consisting of an amorphous matrix embedded with BCC nanocrystals (~10 nm in diameter).
2. Raman spectroscopy confirmed the formation of an amorphous carbon (a-C) phase within the films for carbon-containing samples.
3. The wear rate decreased exponentially from 4.8 × 10^−5^ mm^3^/N·m (0 at.% C) to 6.7 × 10^−6^ mm^3^/N·m (47.6 at.% C). The friction coefficient stabilized at lower values (~0.3 and ~0.2) with carbon incorporation.
4. The corrosion current density (I_corr_) in a 3.5% NaCl solution was reduced by two orders of magnitude, reaching a minimum of 7.16 × 10^−8^ A/cm^2^ for the film with the highest carbon content (47.6 at.%).
5. EIS measurements showed a systematic increase in charge transfer resistance (R_ct_) with carbon content, reaching a maximum of 1,062,900 Ω·cm^2^ for the 5 sccm sample.

The observed microstructural evolution is attributed to the limited solubility of carbon in the BCC lattice, leading to the formation of a secondary amorphous phase. The dramatic improvement in tribological performance is interpreted as a synergistic effect of the solid-lubricating a-C phase and the strengthening role of the BCC nanocrystals. The superior corrosion resistance is a direct result of the dense, defect-poor amorphous network that effectively blocks diffusion pathways for corrosive ions. A critical trade-off was identified: while higher carbon content enhances wear and corrosion resistance, it also induces embrittlement, leading to spalling at 47.6 at.% C. Therefore, an optimal carbon content of ~23 at.% is proposed, offering a balanced combination of properties. This work demonstrates that carbon content is a powerful design parameter for tailoring HEA films, providing a pathway to develop multifunctional protective coatings for extreme environments in sectors such as marine engineering and energy systems.

## Figures and Tables

**Table 1:** Deposition parameters of the (AlFeCoNi)C_x_ HECFs.

Process Parameter	Values
Base pressure (Pa)	1 × 10^−3^
Working pressure (Pa)	0.9
HPPMS power (V-μs-Hz)	800-150-200
Argon flow (sccm)	40
Acetylene flow (sccm)	0, 3, 5
Deposition time (min)	30
Deposition temperature	Room temperature
Substrate bias voltage (V)	−50
Average power density (W/cm^2^)	35.4, 33.7, 45.4
Peak power density (kW/cm^2^)	2.1, 2.1, 2.8
Deposition rate (nm/min)	38, 24, 22
Substrate-target distance (mm)	80

**Table 2:** The corrosion potential (E_corr_) and corrosion current density (I_corr_) for the HECF-coated and uncoated 304 SS.

Samples	304 SS	0 sccm	3 sccm	5 sccm
E_corr_ (V_SCE_)	−0.004	−0.222	−0.045	−0.004
I_corr_ (A/cm^2^)	1.23 × 10^−7^	1.62 × 10^−6^	4.03 × 10^−7^	7.16 × 10^−8^

**Table 3:** Electrochemical impedance parameters obtained by fitting EIS data of the (AlFeCoNi)C_x_ HECFs and 304 SS substrate.

Samples/ Parameter	R_s_ (Ω·cm^2^)	R_p_ (Ω·cm^2^)	CPE_p_ (s^n^·MΩ^−1^·cm^−2^)	R_ct_ (Ω·cm^2^)	CPE_ct_ (sn·MΩ^−1^·cm^−2^)	χ^2^
0 sccm	5.3	1110	20	98,399	10.1	0.0030
3 sccm	5.4	1154	5.2	174,170	11.0	0.0026
5 sccm	4.9	1831	8.4	1,062,900	4.4	0.0203
304 SS	4.8	-	-	534,500	110	0.0045

## Data Availability

The original contributions presented in this study are included in the article. Further inquiries can be directed to the corresponding authors.

## Abbreviations

The following abbreviations are used in this manuscript:

HECFs	High-entropy alloy carbide films
HEAFs	High-entropy alloy films
HPPMS	High-power pulsed magnetron sputtering
EDS	Energy dispersive spectroscopy
XRD	X-ray diffraction
TEM	Transmission electron microscopy
FE-SEM	Field emission scanning electron microscope
EIS	Electrochemical impedance spectroscopy
APT	Atom probe tomography

## References

[1] Chen Y.-Y., Hung S.-B., Wang C.-J., Wei W.-C., Lee J.-W. (2019). High temperature electrical properties and oxidation resistance of V-Nb-Mo-Ta-W high entropy alloy thin films. Surf. Coat. Technol..

[2] Pogrebnjak A.D., Yakushchenko I.V., Bondar O.V., Beresnev V.M., Oyoshi K., Ivasishin O.M., Amekura H., Takeda Y., Opielak M., Kozak C. (2016). Irradiation resistance, microstructure and mechanical properties of nanostructured (TiZrHfVNbTa)N coatings. J. Alloys Compd..

[3] Xu Y., Li G., Xia Y. (2020). Synthesis and characterization of super-hard AlCrTiVZr high-entropy alloy nitride films deposited by HiPIMS. Appl. Surf. Sci..

[4] Tokarewicz M., Grądzka-Dahlke M. (2021). Review of Recent Research on AlCoCrFeNi High-Entropy Alloy. Metals.

[5] Xu J., Kan L., Li H., Gao X., Zhang W., Wei W., Liu X., Yang W., Sun W., An X. (2025). Annealing Treatment of Al_2_CoCrFeNi High-Entropy Alloys: Synergistic Effect of Microstructure Modulation on Mechanical and Thermoelectric Properties. Coatings.

[6] Goodelman D.C., White D.E., Hodge A.M. (2021). Phase transition zones in compositionally complex alloy films influenced by varying Al and Ti content. Surf. Coat. Technol..

[7] Wu J., Wang F., Guo Y., Shang X., Zhang J., Liu Q. (2023). Cu assisted improvement of wear and corrosion resistance in FeCoNiAl high-entropy intermetallic coating by laser cladding. J. Mater. Res. Technol..

[8] Jansson U., Lewin E. (2019). Carbon-containing multi-component thin films. Thin Solid Film..

[9] Yan X., Zhu B., Zhang Y., Guo S., Qiu H. (2024). Phase formation and unusual interstitial solid-solution strengthening behavior of (CoCrFeMnNi)N_x_ high-entropy ceramic films. Surf. Coat. Technol..

[10] Li R., Huang C., Zhou T., Zhang R. (2025). Research on VNbMoTaW-C high entropy alloy carbide films on 316L stainless steel as bipolar plates in proton exchange membrane fuel cells environment. J. Power Sources.

[11] Fritze S., Malinovskis P., Riekehr L., von Fieandt L., Lewin E., Jansson U. (2018). Hard and crack resistant carbon supersaturated refractory nanostructured multicomponent coatings. Sci. Rep..

[12] Wu B.H., Wang Y., Yu Y., Jiang F., Sun H., Jing F.J., Zhu S., Wu Y., Leng Y.X., Huang N. (2015). Modulate the deposition rate through changing the combination of frequency and pulse width at constant duty cycle. Surf. Coat. Technol..

[13] Stasiak T., Debnárová S., Lin S., Koutná N., Czigány Z., Balázsi K., Buršíková V., Vašina P., Souček P. (2024). Synthesis and characterization of ceramic high entropy carbide thin films from the Cr-Hf-Mo-Ta-W refractory metal system. Surf. Coat. Technol..

[14] Jing P.P., Ma D.L., Gong Y.L., Luo X.Y., Zhang Y., Weng Y.J., Leng Y.X. (2021). Influence of Ag doping on the microstructure, mechanical properties, and adhesion stability of diamond-like carbon films. Surf. Coat. Technol..

[15] Deng Q.Y., Wang C.M., Zhang T.F., Yang W., Li X., Huang N., Leng Y.X. (2019). Regulating the uniformity of DLC films in ECR plasma with negative substrate biasing. Surf. Coat. Technol..

[16] Arif M., Eisenmenger-Sittner C. (2017). In situ assessment of target poisoning evolution in magnetron sputtering. Surf. Coat. Technol..

[17] Güttler D., Abendroth B., Grötzschel R., Möller W., Depla D. (2004). Mechanisms of target poisoning during magnetron sputtering as investigated by real-time in situ analysis and collisional computer simulation. Appl. Phys. Lett..

[18] Ma Y., Yang J., Tian X., Gong C., Zheng W., He Y., Gao Z., Wei L., Chu P.K., Zhang K. (2020). Influence of Acetylene on Ti Target Poisoning During Pulse-Enhanced Vacuum Arc Evaporation. IEEE Trans. Plasma Sci..

[19] Cai Y.P., Wang G.J., Ma Y.J., Cao Z.H., Meng X.K. (2019). High hardness dual-phase high entropy alloy thin films produced by interface alloying. Scripta Mater..

[20] Wang X., An Z., Cai J., Jiang C., Su H., Luo X., Li Z., Wu S., Yang L., Long H. (2023). Design of novel AlCoFeNiV high-entropy alloys with high-strength and high-ductility. Mater. Charact..

[21] Qi J., Fan X., Hoyos D.I., Widom M., Liaw P.K., Poon J. (2024). Integrated design of aluminum-enriched high-entropy refractory B2 alloys with synergy of high strength and ductility. Sci. Adv..

[22] Jiang X., Liu W., Huang J., Leng Y. (2025). Achieving superior wear and corrosion resistance in FeCoNiCrNC films via nitrogen and carbon dual anions incorporation. Appl. Surf. Sci..

[23] Takeuchi A., Inoue A. (2005). Classification of Bulk Metallic Glasses by Atomic Size Difference, Heat of Mixing and Period of Constituent Elements and Its Application to Characterization of the Main Alloying Element. Mater. Trans..

[24] Chen S., Aitken Z.H., Pattamatta S., Wu Z., Yu Z.G., Banerjee R., Srolovitz D.J., Liaw P.K., Zhang Y.-W. (2021). Chemical-Affinity Disparity and Exclusivity Drive Atomic Segregation. Short-Range Ordering, and Cluster Formation in High-Entropy Alloys. Acta Mater..

[25] Liu C., Li Z., Lu W., Bao Y., Xia W., Wu X., Zhao H., Gault B., Liu C., Herbig M. (2021). Reactive wear protection through strong and deformable oxide nanocomposite surfaces. Nat. Commun..

[26] Lou B.-S., Lin Y.-C., Lee J.-W. (2023). Mechanical properties and corrosion resistance of AlCrNbSiTiN high entropy alloy nitride coatings. Coatings.

[27] Zheng S., Cai Z., Pu J., Zeng C., Chen S., Chen R., Wang L. (2019). A feasible method for the fabrication of VAlTiCrSi amorphous high entropy alloy film with outstanding anti-corrosion property. Appl. Surf. Sci..

[28] Zheng S., Cai Z., Pu J., Zeng C., Wang L. (2021). Passivation behavior of VAlTiCrSi amorphous high-entropy alloy film with a high corrosion-resistance in artificial sea water. Appl. Surf. Sci..

[29] Feng X.B., Fu W., Zhang J.Y., Zhao J.T., Li J., Wu K., Liu G., Sun J. (2017). Effects of nanotwins on the mechanical properties of Al_x_CoCrFeNi high entropy alloy thin films. Scripta Mater..

[30] Peng H.-E., Lee C.-Y., Chang H.-Y., Yeh J.-W. (2023). Effect of Substrate Bias on the Microstructure and Properties of Non-Equimolar (AlCrSiTiZr)N Films with Different Cr/Zr Ratios Deposited Using Reactive Direct Current Magnetron Sputtering. Coatings.

[31] Liu Y., Xiang D., Wang K., Yu T. (2022). Corrosion of Laser Cladding High-Entropy Alloy Coatings: A Review. Coatings.

[32] Liu M., Chen X.M., Li Y.T., Zeng X.K., Jiang X., Leng Y.X. (2023). HiPIMS deposition of CuNiTiNbCr high-entropy alloy films: Influence of the pulse width on structure and properties. Vacuum.

[33] Nowak W.J., Kubaszek T., Gradzik A., Grądzka-Dahlke M., Perkowski D., Tokarewicz M., Walczak M., Szala M. (2025). Effect of Ti Doping of Al0.7CoCrFeNi-Based High Entropy Alloys on Their Erosion Resistance by Solid Particles. Materials.

[34] Shi X., Liang H., Li Y. (2025). Effect of Si Content on Phase Structure, Microstructure, and Corrosion Resistance of FeCrNiAl_0.7_Cu_0.3_Six High-Entropy Alloys in 3.5% NaCl Solution. Coatings.

